# Structural and functional effects of myosin-binding protein-C phosphorylation in heart muscle are not mimicked by serine-to-aspartate substitutions

**DOI:** 10.1074/jbc.AC118.004816

**Published:** 2018-08-06

**Authors:** Thomas Kampourakis, Saraswathi Ponnam, Yin-Biao Sun, Ivanka Sevrieva, Malcolm Irving

**Affiliations:** From the Randall Centre for Cell and Molecular Biophysics and British Heart Foundation Centre of Research Excellence, School of Basic and Medical Biosciences, King's College London, London SE1 1UL, United Kingdom

**Keywords:** myosin, cardiac muscle, protein phosphorylation, muscle, phosphorylation, cardiac muscle regulation, myofibril, myosin-binding protein-C, phosphomimetic, polarized fluorescence muscle fiber, tris-phosphorylation

## Abstract

Myosin-binding protein-C (cMyBP-C) is a key regulator of contractility in heart muscle, and its regulatory function is controlled in turn by phosphorylation of multiple serines in its m-domain. The structural and functional effects of m-domain phosphorylation have often been inferred from those of the corresponding serine-to-aspartate (Ser–Asp) substitutions, in both *in vivo* and *in vitro* studies. Here, using a combination of *in vitro* binding assays and *in situ* structural and functional assays in ventricular trabeculae of rat heart and the expressed C1mC2 region of cMyBP-C, containing the m-domain flanked by domains C1 and C2, we tested whether these substitutions do in fact mimic the effects of phosphorylation. *In situ* changes in thin and thick filament structure were determined from changes in polarized fluorescence from bifunctional probes attached to troponin C or myosin regulatory light chain, respectively. We show that both the action of exogenous C1mC2 to activate contraction in the absence of calcium and the accompanying change in thin filament structure are abolished by tris-phosphorylation of the m-domain, but unaffected by the corresponding Ser–Asp substitutions. The latter produced an intermediate change in thick filament structure. Both tris-phosphorylation and Ser–Asp substitutions abolished the interaction between C1mC2 and myosin sub-fragment 2 (myosin S2) *in vitro*, but yielded different effects on thin filament binding. These results suggest that some previous inferences from the effects of Ser–Asp substitutions in cMyBP-C should be reconsidered and that the distinct effects of tris-phosphorylation and Ser–Asp substitutions on cMyBP-C may provide a useful basis for future studies.

## Introduction

**Figure 1.**
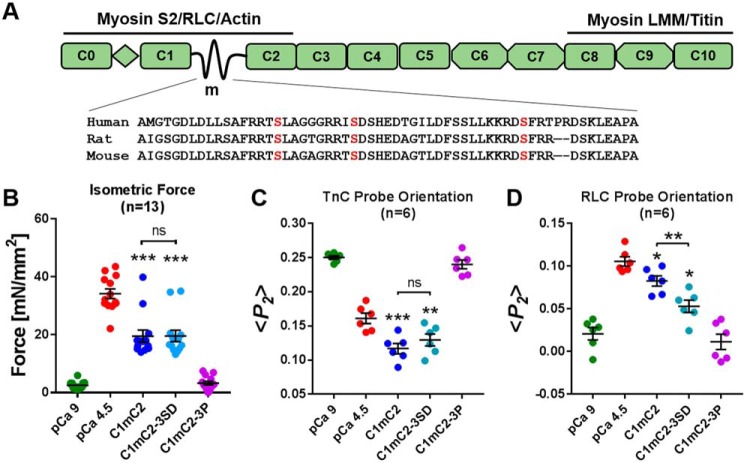
**Effect of phosphomimetic substitutions in cMyBP-C on isometric force and thin and thick filament structure in cardiac muscle.** *A,* schematic of cMyBP-C's domain organization and known protein interactions, with a sequence alignment for the cardiac-specific m-motif containing the phosphorylatable serine residues. *LMM,* light meromyosin; *RLC,* regulatory light chain; *myosin S2,* myosin sub-fragment 2. *B,* isometric force. *C,* TnC probe orientation. *D,* RLC probe orientation of ventricular trabeculae in relaxing conditions (*p*Ca 9, *green*), at full calcium activation (*p*Ca 4.5, *red*), and during incubation in relaxing solution (*p*Ca 9) containing 40 μmol/liter C1mC2 (*blue*), phosphomimetic C1mC2–3SD (*turquoise*), or PKA tris-phosphorylated C1mC2–3P (*purple*). Mean ± S.E. with the number of trabeculae (*n*) indicated in each panel. Statistical significance of differences between groups was assessed with a one-way ANOVA followed by Tukey's post hoc test: *, *p* < 0.05; **, *p* < 0.01; ***, *p* < 0.001, and *ns,* not significant.

Phosphorylation of cardiac myosin-binding protein-C (cMyBP-C)^2^ is an important determinant of cardiac muscle function, and ablation of cMyBP-C phosphorylation has frequently been associated with heart failure, further underlining its functional significance (1). cMyBP-C is phosphorylated *in vivo* (2) and is a substrate for multiple protein kinases that target conserved serine residues in the m-domain or m-motif that link the C1 and C2 Ig domains (Fig. 1*A*) (3). Phosphorylation of these residues controls the interaction of the N-terminal region of cMyBP-C with both the myosin-containing thick and the actin-containing thin filaments (4). Interactions of cMyBP-C's N-terminal domains with myosin are thought to have mainly inhibitory effects on contractility by stabilizing the thick filament OFF state, characterized by myosin head domains bound to the filament core in quasi-helical tracks (5, 6). In contrast, cMyBP-C–actin interactions have been frequently associated with an activating effect on contractility and thin filament structure (5, 7). Phosphorylation of cMyBP-C has been shown to abolish these regulatory interactions and thereby modulate cardiac contractility via changes in the thick and thin filament regulatory state.

Serine to aspartate (Ser–Asp) substitutions of serine residues within the m-motif have often been used as a proxy for phosphorylation, *in vivo* in transgenic animal models (8, 9), *in situ* in isolated cardiac muscle cells (10), and *in vitro* in isolated proteins (11, 12). The assumption that Ser–Asp substitutions reproduce the effects of phosphorylation in cMyBP-C has generally not been critically examined. *In vivo* experiments on transgenic animal lines do not allow direct comparison of the effects of fully phosphorylated and Ser–Asp substituted cMyBP-C on cardiac muscle function. In contrast, *in vitro* experiments using isolated proteins allow some functional mechanisms to be characterized at the molecular level, but they do not reproduce regulatory and contractile mechanisms that depend on the native lattice of thick and thin filaments.

In this study, we used bifunctional fluorescent probes on myosin regulatory light chain (RLC) or troponin C (TnC) to monitor structural changes in the thick and thin filaments, respectively, in rat heart muscle cells. We induced activation of the muscle cells using exogenous rat cardiac C1mC2 fragments that were native, tris-phosphorylated, or carrying three Ser–Asp substitutions. We combined these structural measurements with measurements of active isometric force and with biochemical characterization of the interaction between C1mC2 and myosin S2, and C1mC2 and native thin filaments (NTF) *in vitro*, to determine whether Ser–Asp substitutions reproduce the structural and functional effects of phosphorylation.

## Results

### Phosphomimetic substitutions in C1mC2 do not abolish thin filament activation

N-terminal fragments of rat cardiac myosin-binding protein-C containing the Ig-domains C1 and C2 and the intervening m-domain (C1mC2) are sufficient to activate contraction of demembranated rat ventricular trabeculae in the absence of Ca^2+^, and tris-phosphorylation of C1mC2 by protein kinase A (PKA) abolishes this effect (5). This assay therefore enables a test of the ability of Ser–Asp substitutions to act as phosphomimetics for this mode of action of cMyBP-C. Trabeculae were incubated in relaxing solution (*p*Ca 9) containing 40 μmol/liter unphosphorylated C1mC2, PKA tris-phosphorylated C1mC2 (C1mC2–3P; Fig. S1), or C1mC2 with three phosphorylatable serines mutated to aspartate (C1mC2–3SD) (Fig. 1). Incubation in C1mC2 produced an isometric force of 57 ± 5% (mean ± S.E., *n* = 13) of that during full Ca^2+^ activation (Fig. 1*B*), whereas C1mC2–3P produced no activation, as described previously (5). Strikingly, C1mC2–3SD activated contraction to the same extent as C1mC2 (57 ± 5%, mean ± S.E., *n* = 13). In complete contrast to phosphoserines, the Ser–Asp substitutions have no effect on the activating effect of C1mC2.

We used bifunctional (sulfo)rhodamine probes attached to either the E-helix of cardiac troponin C (BR–cTnC-E) or cross-linking helices B and C of cardiac myosin regulatory light chain (BSR–cRLC-BC) (Fig. S2) to monitor structural changes in the thin and thick filaments, respectively, in this assay. The cTnC E-helix is part of the core domain of troponin containing troponin I and troponin T (IT-arm of troponin), and its orientation is coupled to the position of tropomyosin on the surface of the thin filament. The order parameter 〈*P*_2_〉 describing the orientation of BR–cTnC-E decreases on calcium activation, suggesting a more perpendicular orientation of the E-helix (Fig. 1*C*). Incubation of trabeculae in relaxing solution (*p*Ca 9) containing 40 μmol/liter C1mC2 induced a change in thin filament structure reported by the TnC E-helix probe that is significantly larger than that during full calcium activation alone, as reported previously (5). In contrast with unphosphorylated C1mC2, C1mC2–3P had no effect on thin filament structure. However, C1mC2–3SD had the same effect as C1mC2 (Fig. 1*C*). Thus, in full agreement with the functional effects described above, the Ser–Asp substitutions do not reproduce the effect of tris-phosphorylation of C1mC2 on thin filament structure.

The RLC BC-helix probe is mainly sensitive to the regulatory state of the thick filament (13), and 〈*P*_2_〉 for this probe increases on calcium activation (Fig. 1*D*). Incubation of BSR–cRLC–BC exchanged trabeculae in 40 μmol/liter C1mC2 induced a change in BSR–cRLC–BC probe orientation of ∼70% of that for calcium activation, whereas C1mC2–3P produced no significant change in probe orientation. C1mC2–3SD had an intermediate effect.

The above results show that the Ser–Asp substitutions in C1mC2 do not mimic the effects of tris-phosphorylation for both active force and accompanying effects on thin and thick filament structure. For active force and thin filament structure, the action of C1mC2–3SD was indistinguishable from that of the native fragment. For thick filament structure, the effect was intermediate, suggesting the possibility that Ser–Asp substitutions in the m-domain might mimic phosphorylation of these residues with respect to the direct action of C1mC2 on thick but not thin filament structure, in the light of previous evidence for coupling between the regulatory states of the thin and thick filaments (5, 14).

### Phosphomimetic substitutions in C1mC2 alter thin filament interaction and abolish binding to myosin S2

**Figure 2.**
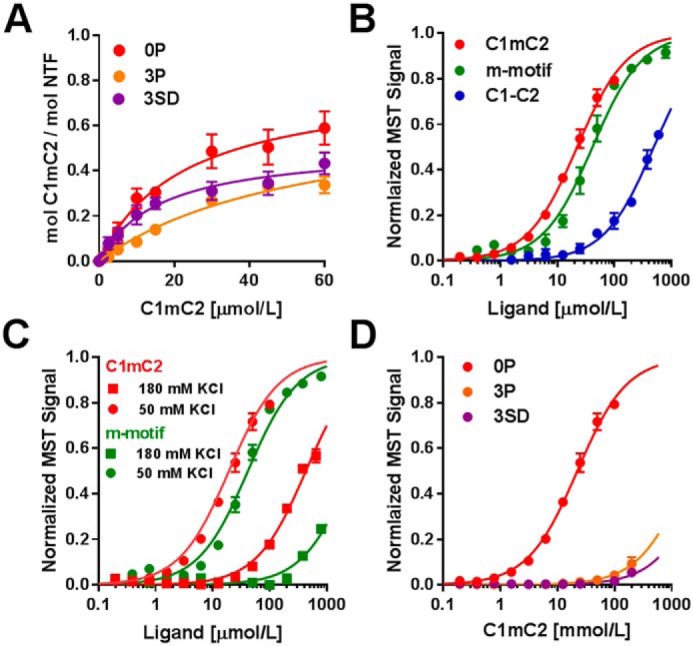
**Binding of cMyBP-C N-terminal domains to NTF and myosin S2Δ.** *A,* NTF co-sedimentation binding data for C1mC2 (*red*), C1mC2–3P (*orange*), and C1mC2–3SD (*purple*). *B,* normalized MST binding curves for C1mC2 (*red*), isolated m-motif (*green*), or C1–C2 (*blue*) titrated against myosin S2Δ. *C,* normalized MST curves for C1mC2 (*red*) and m-motif (*green*) binding to myosin S2Δ in the presence of 50 mm KCl (*circles*) and 180 mm KCl (*squares*). *D,* binding of C1mC2 (*red*), C1mC2–3P (*orange*), and C1mC2–3SD (*purple*) to myosin S2Δ. For all experiments, means ± S.E., *n* = 4–8.

We used high-velocity co-sedimentation to determine the effects of phosphorylation and Ser–Asp substitutions in C1mC2 on binding to isolated NTF. The unphosphorylated C1mC2 binds NTFs in a saturable manner with a *K_d_* of 23 ± 7 μmol/liter (mean ± S.E., *n* = 7) and a *B*_max_ of 0.81 ± 0.10 mol/mol, suggesting roughly stoichiometric binding of C1mC2 to actin (Fig. 2*A*). PKA tris-phosphorylation of C1mC2 increased *K_d_* to 57 ± 5 μmol/liter (mean ± S.E., *n* = 8) and reduced *B*_max_ to 0.67 ± 0.06 mol/mol, indicating reduced binding to NTFs *in vitro*, in agreement with previously published results (15). C1mC2–3SD (Fig. 2*A*, *purple*) binds NTFs with a *K_d_* of 16 ± 5 μmol/liter (mean ± S.E., *n* = 4), similar to that of unphosphorylated C1mC2, but with a significantly lower *B*_max_, 0.51 ± 0.06 mol/mol.

Next, we tested whether Ser–Asp substitutions mimic the effect of phosphorylation of m-domain serines on the binding of C1mC2 to myosin S2Δ, the first 126 amino acids of myosin S2 (16), by microscale thermophoresis (MST). Binding of C1mC2 to myosin S2Δ is strongly temperature-dependent. At 21 °C the *K_d_* was ∼100 μmol/liter, but at 30 °C it was reduced to ∼20 μmol/liter, in agreement with previous results (Fig. S3; Table 1) (16). Moreover, the binding curve is biphasic, indicating an additional low-affinity binding interaction with an estimated *K_d_* of ∼400 μmol/liter (Fig. S3, *arrowhead*).

**Table 1:** **Summary of equilibrium dissociation constants (*K_d_*) for different cMyBP-C constructs for myosin S2Δ as measured by microscale thermophoresis** Means are ± S.E. (*n* = 4–6). cd − *K_d_* cannot be reliably determined.

Ligand	*T*	KCl	*K_d_*
	°*C*	*mmol/liter*	μ*mol/liter*
C1mC2	21	50	106.2 ± 11.2
	25	50	58.5 ± 8.5
	30	50	21.3 ± 3.5
	30	180	364.5 ± 10.6
C1–C2	30	50	477.4 ± 6.7
m-motif	30	50	41.1 ± 2.3
	30	180	cd
C1mC2–3P	30	50	cd
C1mC2–3SD	30	50	cd

As a step toward localizing the binding interactions between cMyBP-C and myosin S2Δ, we measured the binding affinity for myosin S2Δ to the isolated m-motif and to a construct containing Ig domains C1 and C2 connected by a flexible linker (C1–C2), *i.e.* with the m-motif deleted (Fig. 2*B*; Table 1). The m-motif alone binds myosin S2Δ with a *K_d_* of ∼40 μmol/liter, indicating that it is the main interaction site between C1mC2 and myosin S2Δ. In contrast C1–C2 binds myosin S2Δ with a *K_d_* of ∼500 μmol/liter, suggesting that the flanking C1 and C2 domains are responsible for the low-affinity binding sites in C1mC2 and only have a small contribution to C1mC2 binding to myosin S2Δ.

Increasing ionic strength greatly reduced the affinity of C1mC2 for myosin S2Δ (Fig. 2*C*; Table 1), suggesting that the interaction between the m-motif and myosin S2Δ is mainly ionic. Consistent with that interpretation, binding of the isolated m-motif to myosin S2Δ was largely abolished at the higher ionic strength.

Both PKA tris-phosphorylation and replacement of the three phosphorylatable serines by aspartate abolished the high-affinity interaction between C1mC2 and myosin S2Δ (Fig. 2*D,* Fig. S4, and Table 1), as expected for the largely ionic nature of the high-affinity binding site in the m-motif.

## Discussion

The results presented above show that serine-to-aspartate substitution of the three phosphorylation sites in the m-motif of cMyBP-C fully mimic the effects of phosphorylation of those sites with respect to binding to myosin S2Δ but fail to mimic its effects on the interaction between cMyBP-C and the thin filament. The ionic strength dependence of the binding between cMyBP-C and myosin S2Δ suggests that it is mainly driven by ionic interactions between the m-motif and myosin S2Δ, consistent with the fact that it can be abolished by the introduction of additional negative charges, by either phosphorylation or Ser–Asp substitutions. In contrast, regulatory interactions of cMyBP-C with NTFs have been localized to both domain C1 and the m-motif (17). Phosphorylation of the m-motif serines appears to control these regulatory interactions by altering C1mC2 structure (18), in a way that is not efficiently mimicked by Ser–Asp substitutions. Although Ser–Asp substitutions reduce the binding capacity of C1mC2 to NTFs to a similar extent as that observed for the effects of PKA phosphorylation on the native protein, they do not affect *K_d_* and thin filament activation, suggesting that phosphorylation might affect thin filament activation via a structural change communicated between the m-motif and domain C1 (15).

**Figure 3.**
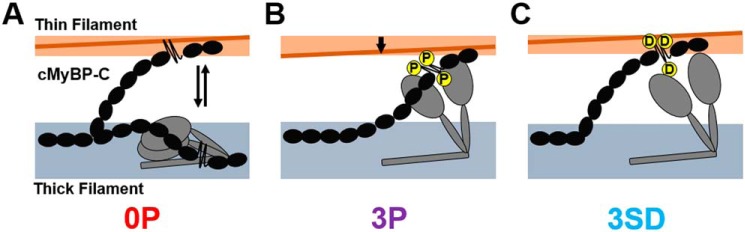
**Model for the effect of phosphorylation or serine-aspartate substitutions on cMyBP-C function.** *A,* in the unphosphorylated state, cMyBP-C binds both thin and thick filaments stabilizing their ON and OFF states, respectively. cMyBP-C inhibits myosin head domains (*gray*) by stabilizing their folded OFF state and activates the thin filament by moving tropomyosin (*brown*) away from its blocked position toward the open position. *B,* PKA tris-phosphorylation (*P*) abolishes both the activating and inhibitory effects of cMyBP-C on the thin and thick filament, respectively. *C,* in contrast, serine-to-aspartate substitutions (*D*) abolish the inhibitory interaction between cMyBP-C and myosin but do not abolish the activating interaction with the thin filament.

These results in combination with previous work therefore suggest the following: unphosphorylated native cMyBP-C binds to both the thin and thick filaments (Fig. 3*A*), stabilizing their ON and OFF states, respectively; cMyBP-C phosphorylation abolishes both regulatory interactions, so that myosin heads are released from the thick filament backbone but the thin filament is OFF (Fig. 3*B*); and substitution of the phosphorylatable serines by aspartates releases the myosin heads from the thick filament backbone without affecting the regulatory interaction with the thin filament (Fig. 3*C*), thereby stabilizing the ON state of both thin *and* thick filaments. It follows that it could be quite misleading to interpret the effects of Ser–Asp substitutions in cMyBP-C as reproducing the structural or functional effects of tris-phosphorylation of cMyBP-C *in vitro* or *in vivo*. However, the differential effects of Ser–Asp substitutions and tris-phosphorylation on the thin and thick filament interactions of cMyBP-C may provide a useful approach to dissect the effects of those interactions in future studies.

## Experimental procedures

### Preparation of bifunctional rhodamine-labeled cRLC and cTnC, and exchange into demembranated rat ventricular trabeculae

Wistar rats (male, 200–250 g) were sacrificed by cervical dislocation (Schedule 1 procedure in accordance with UK Animal Scientific Procedure Act, 1986), and demembranated right ventricular trabeculae were prepared as described previously (19). All procedures were carried out in accordance with the guidelines of the Animal Welfare and Ethical Review Body (AWERB, King's College London). All animals were treated in accordance with the guidelines approved by the UK Animal Scientific procedures Act (1986) and European Union Directive 2010/63/EU.

BSR-labeled cRLCs and BR-labeled TnCs were prepared as described previously (5, 19). BSR–cRLCs were exchanged into demembranated trabeculae by extraction in CDTA/rigor solution (composition in mmol/liter: 5 CDTA, 50 KCl, 40 Tris-HCl, 0.1% (v/v) Triton X-100, pH 8.4) for 30 min followed by reconstitution with 40 μmol/liter BSR–cRLC in relaxing solution for 1 h at 22 °C, replacing ∼50% of the endogenous RLC (5). BR–cTnCs were exchanged by an overnight soak on ice of demembranated trabeculae in relaxing solution containing 0.5 mg/ml BR–cTnCs, replacing ∼70% of the endogenous TnCs (20).

### Protein preparation and phosphorylation

All proteins were cloned from a rat cDNA library (BioChain) into a modified pET6a vector fused to an N-terminal hexa-histidine tag followed by TEV cleavage site. C1mC2 Ser–Asp mutations were introduced according to previously published protein primary sequences (8) and introduced by site-directed mutagenesis. Protein constructs were expressed in BL21(DE3)-RIPL cells (Agilent Technologies) followed by affinity chromatography on HisTrapFF columns (GE Healthcare) and removal of the tag sequence by TEV protease treatment. Proteins were further purified by ion-exchange chromatography (IEC) on Mono S columns (GE Healthcare), concentrated to >100 μmol/liter, snap-frozen in liquid nitrogen, and stored in aliquots at −80 °C for experiments. Myosin S2Δ was cloned from a rat heart cDNA library (BioChain) into a modified pET6a vector and expressed and purified as described above, except that IEC was instead performed on a Mono Q column. Purity was estimated by SDS-PAGE and ESI-MS to >95%. C1mC2 was phosphorylated by PKA and purified as described previously (5). Briefly, 50 μmol/liter C1mC2 in PKA assay buffer (composition in mmol/liter: 20 Tris-HCl, 50 NaCl, 2 MgCl_2_, 0.1 EDTA, 1 DTT) was incubated with 2000 units/ml of the catalytic subunit of PKA for 30–60 min at 30 °C. The reaction was followed by Phostag^TM^–SDS-PAGE and ESI-MS (Fig. S1). The mainly tris-phosphorylated C1mC2 was further purified by IEC on Mono S, and homogeneity was determined by Phostag^TM^–SDS-PAGE and ESI-MS to be >95%. Prolonged incubation of C1mC2–3P with demembranated trabeculae did not result in any significant protein dephosphorylation (Fig. S1*B*). For experiments, aliquots of proteins were thawed on ice, gel-filtered, and dialyzed against new buffer prior to each experiment.

### Fluorescence polarization experiments

Activation protocols and composition of experimental solutions were identical to those described previously for fluorescence polarization experiments (21). Polarized fluorescence intensities were measured as described previously for skeletal and cardiac muscle fibers. Trabeculae were mounted between a strain gauge force transducer (KRONEX, Oakland, CA; model A-801, resonance frequency ∼2 kHz) and motor (Aurora Scientific, Dublin, Ireland; Model 312C). Fluorescence emission from BSR–cRLCs and BR–cTnCs in trabeculae was collected by a 0.25 N.A. objective using an excitation light beam in line with the emission path. The polarization of the excitation and emitted beams was set either parallel or perpendicular to the trabecular axis, allowing determination of the order parameter 〈*P*_2_〉 that describes the dipole orientations in the trabeculae (22). The sarcomere length of trabeculae was measured by laser diffraction in relaxing solution prior to each activation. Activating solution contained (in mmol/liter): 25 imidazole, 15 Na_2_CrP, 58.7 KPr, 5.65 Na_2_ATP, 6.3 MgCl_2_, 10 CaCl_2_, 10 K_2_EGTA, 1 DTT, pH 7.1. Each activation was preceded by a 2-min incubation in pre-activating solution (composition in mmol/liter: 25 imidazole, 15 Na_2_CrP, 108.2 KPr, 5.65 Na_2_ATP, 6.3 MgCl_2_, 0.2 K_2_EGTA, 1 DTT, pH 7.1). Isometric force and steady-state fluorescence polarization values were measured once steady force had been established.

### Native thin filament co-sedimentation

Native thin filaments (NTF) were prepared according to previously published protocols (23), and co-sedimentation experiments were performed as described in Ref. 15.

### Microscale thermophoresis

Microscale thermophoresis experiments were performed on a Monolith NT.115 instrument (NanoTemper, Cambridge CB3 0AX, UK) in interaction buffer containing 20 mmol/liter MOPS, pH 7, 1 mmol/liter EDTA, 50 mmol/liter KCl, 1 mmol/liter DTT, and 0.05% (v/v) Tween 20. Myosin S2Δ was labeled with Alexa 647–NHS (Molecular Probes Inc., ThermoFisher Scientific, Paisley, UK) according to the manufacturer's instructions, and dye incorporation (efficiency of ∼80%) was confirmed by HPLC and ESI-MS. All proteins were either gel-filtered into and/or extensively dialyzed against interaction buffer. Titration experiments were performed with a fixed Alexa 647–myosin S2Δ concentration of 100 nmol/liter in standard treated capillaries. For experiments with isolated m-motif, the pH of the MST buffer was adjusted to 6.2, and experiments were performed in enhanced gradient standard capillaries.

### Statistical analysis

Statistical significance of the difference between groups was assessed with a one-way ANOVA followed by Tukey's post hoc test. Details of significance levels are shown in the figure legends.

## Author contributions

T. K. and M. I. conceptualization; T. K. and S. P. data curation; T. K. and M. I. formal analysis; T. K. and S. P. investigation; T. K. visualization; T. K. writing-original draft; Y.-B. S. and I. S. resources; Y.-B. S. and I. S. methodology; I. S. and M. I. writing-review and editing.

## Supplementary Material

Supporting Information

## Footnotes

2 The abbreviations used are:

cMyBP-C: cardiac isoform of myosin-binding protein-C
BSR: bifunctional sulforhodamine
C1mC2: fragments of cardiac myosin-binding protein-C containing the Ig-domains C1 and C2 and the intervening m-domain
cRLC: cardiac isoform of myosin regulatory light chain
cTnC: cardiac isoform of troponin C
PKA: protein kinase A or cAMP-dependent protein kinase
NTF: native thin filament
ANOVA: analysis of variance
MST: microscale thermophoresis
BR: bifunctional rhodamine
CDTA: 1,2-cyclohexylenedinitrilotetraacetic acid
IEC: ion-exchange chromatography
TEV: tobacco etch virus
ESI: electrospray ionization.
