# Supplementary material for: Structural and functional effects of myosin-binding protein-C phosphorylation in heart muscle are not mimicked by serine-to-aspartate substitutions
Source: J Biol Chem. 2018 Aug 6;293(37):14270–5. doi: 10.1074/jbc.AC118.004816 (PMC6139572; doi:10.1074/jbc.AC118.004816)
Supplement: Supporting Information [file supp_293_37_14270__index.html]

Structural and functional effects of myosin binding protein-C phosphorylation in heart muscle are not mimicked by serine-to-aspartate substitutions — Serine-to-aspartate substitutions in cMyBP-C — Structural and functional effects of myosin-binding protein-C phosphorylation in heart muscle are not mimicked by serine-to-aspartate substitutions — ACCELERATED COMMUNICATION: Ser-to-Asp substitutions in cMyBP-C — Supporting Information 

# Structural and functional effects of myosin-binding protein-C phosphorylation in heart muscle are not mimicked by serine-to-aspartate substitutions

## Supporting Information

- Supporting Information (to be published online) - Supporting Information
