# Supplementary material for: Structural and functional effects of myosin-binding protein-C phosphorylation in heart muscle are not mimicked by serine-to-aspartate substitutions
Source: J Biol Chem. 2018 Aug 6;293(37):14270–5. doi: 10.1074/jbc.AC118.004816 (PMC6139572; doi:10.1074/jbc.AC118.004816)
Supplement: Supporting Information [file supp_AC118.004816_139345_1_supp_180178_pd1b9m.pdf]

Structural and functional effects of myosin binding protein-C phosphorylation in heart muscle are not mimicked by serine-to-aspartate substitutions

Thomas Kampourakis, Saraswathi Ponnam, Yin-Biao Sun, Ivanka Sevrieva, and Malcolm Irving

Randall Centre for Cell and Molecular Biophysics, and British Heart Foundation Centre of Research Excellence, School of Basic and Medical Biosciences, King's College London, SE1 1UL

Supporting Information

Supporting Information Figures and Figure Legends

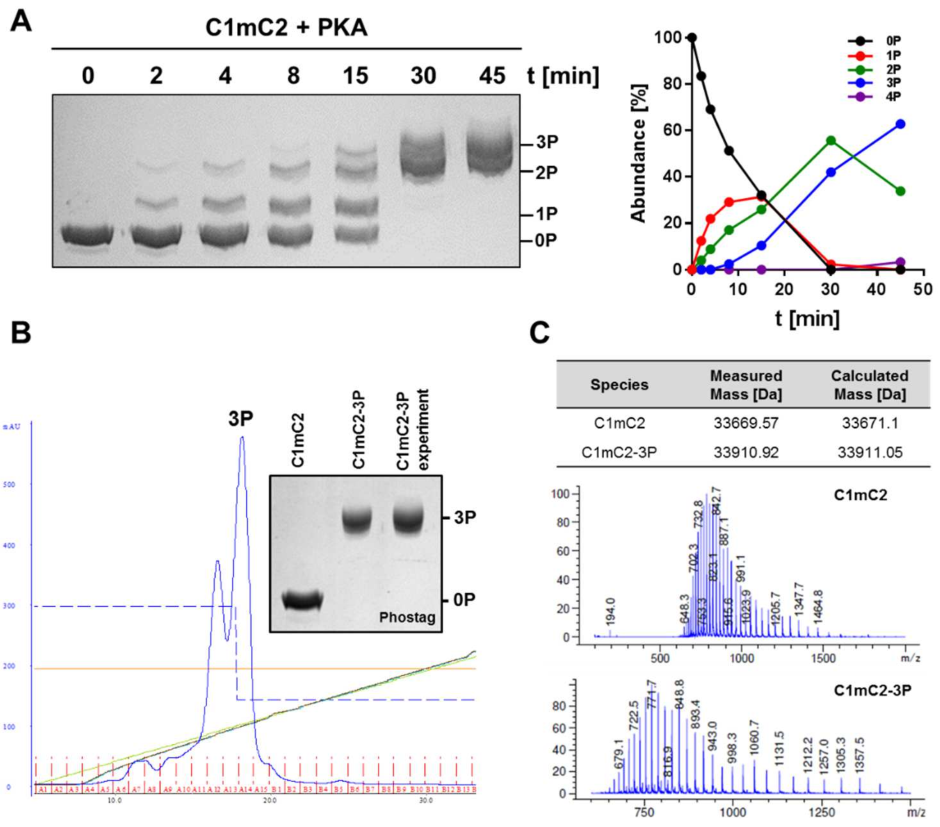

Figure S1. Preparation of PKA tris-phosphorylated C1mC2. (A) Preparative PKA phosphorylation of C1mC2 was followed by Phostag^{TM}-SDS-PAGE (left) and analysed by densitometry (right). Note that Phostag^{TM}-SDS-PAGE clearly separated the individual phospho-species. (B) Chromatogram of C1mC2-3P purified by ion-exchange chromatography. The peak corresponding to the tris-phosphorylated C1mC2 is labelled accordingly. Inset top right: Phostag^{TM}-SDS-PAGE of unphosphorylated C1mC2 and purified PKA tris-phosphorylated C1mC2 (C1mC2-3P), and C1mC2-3P after prolonged incubation (>1h) of C1mC2-3P with demembranated trabeculae. (C) ESI mass spectrum of purified C1mC2 and C1mC2-3P. The measured masses corresponds well with the calculated masses for unphosphorylated and tris-phosphorylated C1mC2.

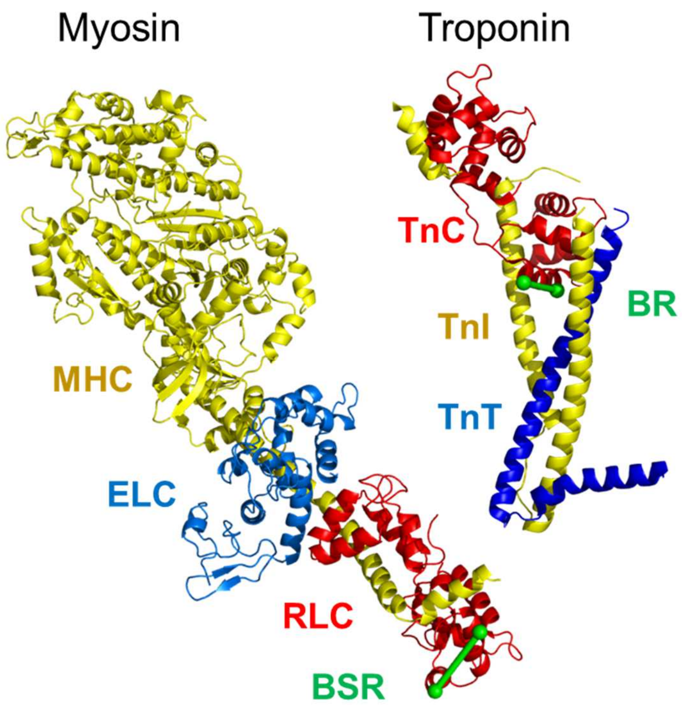

Figure S2. Bifunctional sulfo-rhdoamnine (BSR) and bifunctional rhodamine (BR) labelling positions on the cardiac myosin regulatory light chain (RLC, left) and cardiac troponin C (TnC, right), respectively.

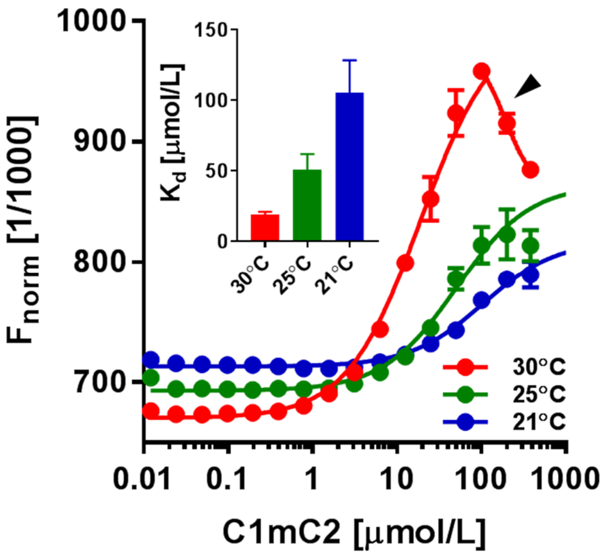

Figure S3. Temperature dependence of C1mC2 binding to myosin S2Δ measured by Microscale Thermophoresis (21°C, blue; 25°C, green; 30°C, red). Inset at the top left shows the calculated dissociation constants (K_d) at the three different temperatures. The arrowhead indicates the inversion of the MST signal at C1mC2 concentrations greater than 100 µmol/L corresponding to a second low affinity binding site of C1mC2 for myosin S2Δ. Mean ± SEM (n=3-4).

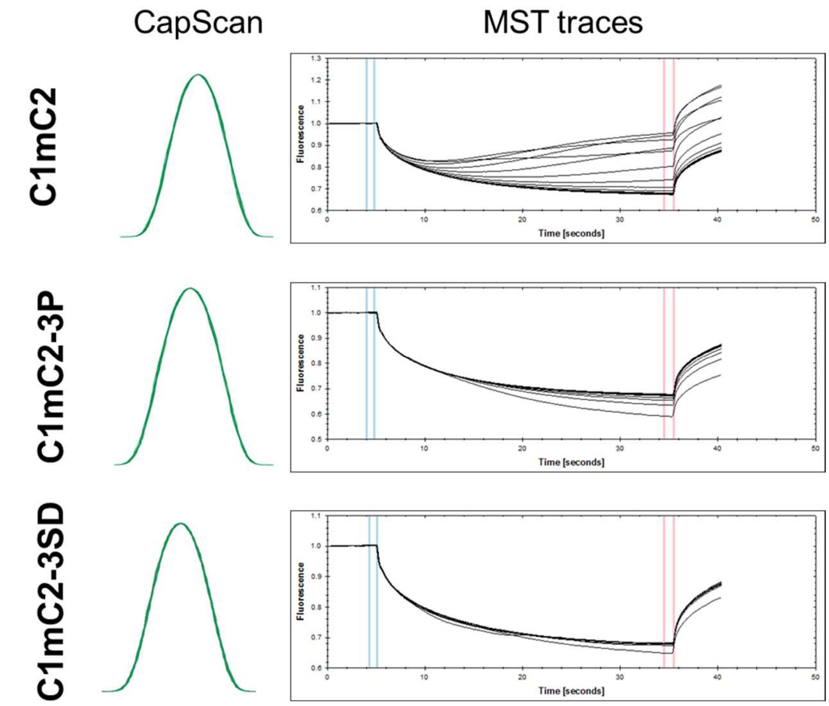

Figure S4. Representative MST traces for C1mC2, C1mC2-3P and C1mC2-3SD binding to myosin S2Δ. The CapScans on the left indicate no unspecific binding of proteins to the capillary walls. The original MST traces (right) show no aberrant behaviour, indicating no protein aggregation.
